# Accuracy of ECV imaging for the detection of subendocardial infarction - comparison with black blood delayed enhancement and pathology

**DOI:** 10.1186/1532-429X-18-S1-Q8

**Published:** 2016-01-27

**Authors:** Elizabeth Jenista, David C Wendell, Han W Kim, Dina Labib, Sung-A Chang, Wolfgang G Rehwald, Enn-Ling Chen, Michele Parker, Raymond Kim

**Affiliations:** 1 grid.26009.3d0000000419367961Duke University, Durham, NC USA; 2 Siemens Healthcare, Chicago, IL USA

## Background

Delineation of diseased and normal tissue is fundamental to identifying cardiac pathology. Studies suggest that parametric extracellular volume fraction (ECV) imaging is superior to conventional delayed enhancement for detection of diffuse, *global* myocardial disease. Conversely, the sensitivity of ECV for the detection of *focal* disorders is unclear. This may be important for subendocardial disease since the standard methodology for ECV requires that "regions of interest.. have adequate margins of separation from tissue interfaces prone to partial volume averaging such as between myocardium and blood" [1]. We have developed a new, **F**low-**I**ndependent **D**ark-blood **D**e**L**ayed **E**nhancement technique (FIDDLE) that increases the conspicuity of subendocardial hyperenhancement, by making the blood pool black [2]. In this study, we compared ECV and FIDDLE for the detection of subendocardial infarction as verified by pathology.

## Methods

Canines (n = 7) underwent variable coronary occlusion to create a range of infarct sizes including 2 sham operated controls. CMR was performed 2-5 days after MI followed immediately by excision and TTC staining to provide a gold standard histopathology reference. ECV was calculated using T1-maps generated with MOLLI sequence (SIEMENS WIP 448B) before and after gadolinium administration (0.2 mmol/kg). FIDDLE images were acquired in identical slice locations 10-20 minutes after gadolinium administration. FIDDLE and ECV analysis were performed separately, blinded to subject identity and pathology. ECV was calculated as previously reported [3]. 26 patients with enzymatically confirmed MI, and 10 normal volunteers with no history of CAD, and a low Framingham risk were enrolled. Imaging and analysis was identical to that for canines. Subendocardial infarction was defined as <50% transmural.

## Results

**Figure 1:**
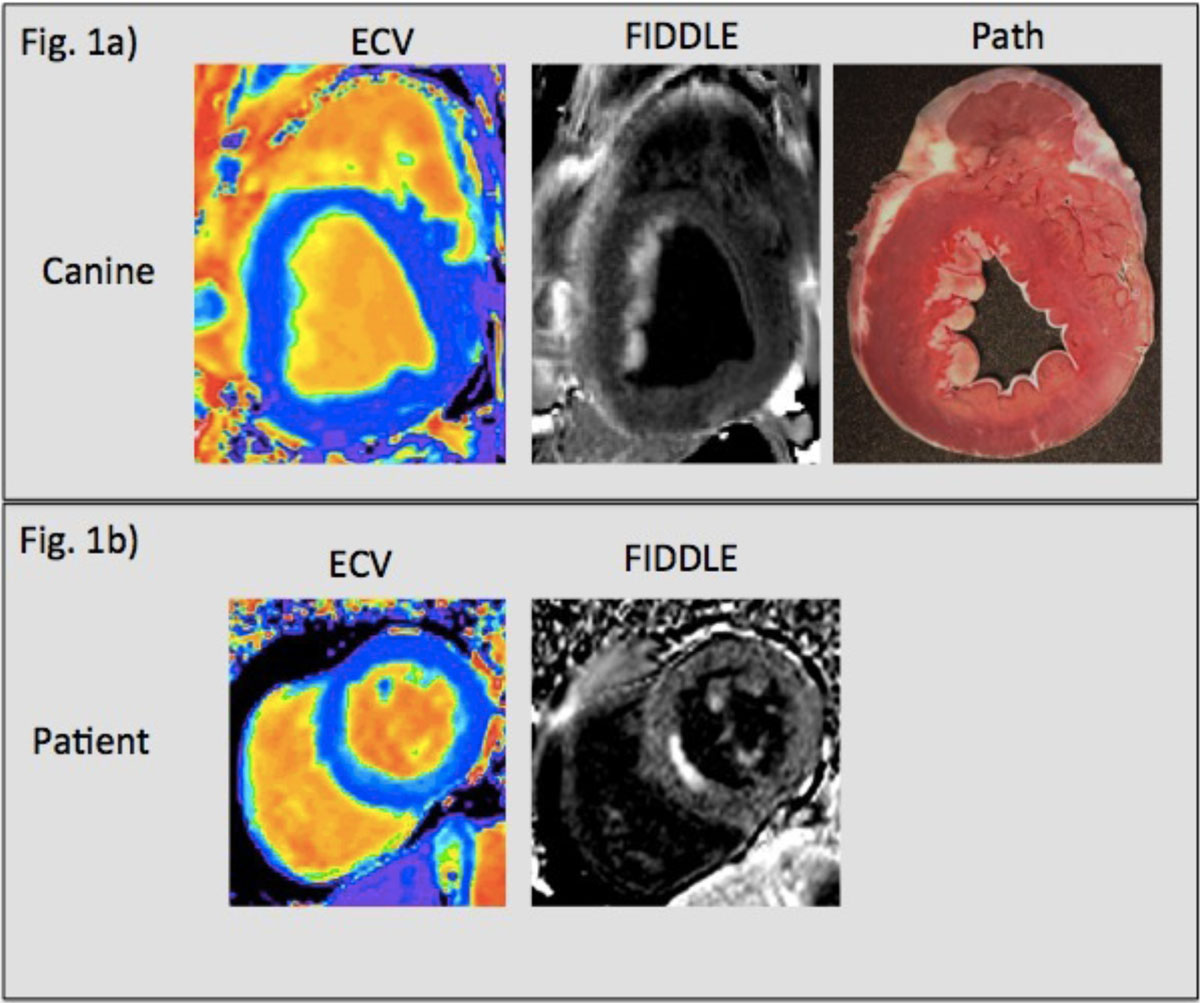
Figure 1

Pathology confirmed MI was found only in animals that underwent coronary occlusion. Mean ECV in controls was 29.8% ± 2.2%, and abnormal ECV was defined as >2SD above the mean (34.2%). Analysis of the 41 matched slices in canines showed that ECV was insensitive for the detection of subendocardial infarcts (3%) while transmural infarcts were routinely detected (91%). Conversely, FIDDLE had high sensitivity for the detection of both subendocardial (93%) and transmural (100%) infarcts. In patients, mean ECV in controls was 26.3% ± 2.6%, and abnormal ECV was (>2SD, 31.9%). Using FIDDLE as the reference standard, ECV was insensitive for the detection of subendocardial MI (26%). Figure 1 shows example images in a canine and patient with subendocardial infarction. Table summarizes the diagnostic performance of ECV and FIDDLE in animals and patients.

## Conclusions

FIDDLE provides excellent visualization and detection of infarcts, while ECV frequently misses subendocardial infarcts. This suggests that ECV imaging is not optimal for the detection of focal, subendocardial disease.

**Table 1:** Diagnostic performance of ECV and FIDDLE in canines and patients.

	Sensitivity	Specificity	Accuracy
Overall - Animals			
FIDDLE	95% (39/41)	100% (170/170)	99% (209/211)
ECV	27% (11/41)	98% (166/170)	84% (177/211)
Subendocardial (<50% transmurality by pathology)			
FIDDLE	93% (28/30)	100% (170/170)	99% (198/200)
ECV	3% (1/30)	98% (166/170)	84% (167/200)
Transmural (>50% transmurality by pathology)			
FIDDLE	100% (11/11)	100% (170/170)	100% (181/181)
ECV	91% (10/11)	98% (166/170)	87% (176/181)
Overall - Patients			
ECV	53% (25/47)	90% (28/31)	68% (53/78)
Subendocardial (<50% transmurality by FIDDLE)			
ECV	26% (5/19)	94% (28/31)	66% (33/50)
Transmural (>50% transmurality by FIDDLE)			
ECV	71% (20/28)	94% (28/31)	83% (48/59)
